# A bibliometric analysis of PCSK9 inhibitors from 2007 to 2022

**DOI:** 10.3389/fendo.2023.1218968

**Published:** 2023-11-29

**Authors:** Qin Luo, Zhenchu Tang, Panyun Wu, Zhangling Chen, Zhenfei Fang, Fei Luo

**Affiliations:** ^1^ Department of Cardiovascular Medicine, The Second Xiangya Hospital, Central South University, Changsha, Hunan, China; ^2^ Research Institute of Blood Lipid and Atherosclerosis, The Second Xiangya Hospital, Central South University, Changsha, Hunan, China; ^3^ Department of Neurology, The Second Xiangya Hospital, Central South University, Changsha, Hunan, China; ^4^ Hunan Key Laboratory of Tumor Models and Individualized Medicine, The Second Xiangya Hospital of Central South University, Changsha, Hunan, China

**Keywords:** PCSK9 inhibitors, cardiovascular endocrinology, bibliometric analysis, data visualization, trend, hotspots

## Abstract

**Background:**

Since the approval of the proprotein convertase subtilisin/kexin type 9 (PCSK9) monoclonal antibodies for marketing in 2015, PCSK9 inhibitors have attracted significant interest in the field of cardiovascular endocrinology. A large number of clinical trials have confirmed the efficacy and safety of PCSK9 inhibitors in reducing cholesterol and the risk of cardiovascular events. No bibliometric analysis of PCSK9 inhibitors has been performed as of yet. This study aims to analyze the research trends and hotspots of PCSK9 inhibitors through bibliometric analysis.

**Methods:**

We searched the Web of Science Core Collection (WoSCC) database for PCSK9 inhibitor-related publications from 2007 to 2022. Data visualization analysis was performed using CiteSpace software. Microsoft Excel and Graphpad software were used for the drawing of some tables and figures.

**Results:**

A total of 1072 pieces of literature were retrieved between 2007 and 2022. The number of publications concerning PCSK9 inhibitors is growing annually. The top five countries with the most articles published were the United States, England, Canada, Italy, and France. Harvard University, Amgen, Brigham & Women’s Hospital, Harvard Medical School, and Imperial College London are the five institutions with the highest output. The *Journal of Clinical Lipidology* is the most popular journal in this field. The most frequently cited journal is the *New England Journal of Medicine*. As for authors, Sabatine MS and Giugliano RP from Brigham & Women’s Hospital have the highest number of published articles. Amgen is the funding agency for most of the research. According to keyword analysis, “low density lipoprotein”, “familial hypercholesterolemia”, “PCSK9 inhibitor”, “PCSK9”, and “efficacy” are the five keywords with the highest frequency of co-occurrence.

**Conclusion:**

The past 15 years have witnessed a rapid and fruitful development of PCSK9 inhibitors. The research trend and focus for PCSK9 inhibitors are from the mechanism of reducing low-density lipoprotein cholesterol to related clinical trials. Developed countries such as the United States have contributed prominently in this area. Coronary artery and inflammation are currently at the forefront of research in the field and are in an explosion period.

## 1 Introduction

Proprotein convertases (PCs) are responsible for cleaving inactive protein precursors to generate bioactive protein products, which will lead to the activation (in most cases) and inactivation of precursor proteins (1–4). Proprotein convertase subtilisin/kexin type 9 (PCSK9), the last member of the PCs family, was first reported in 2003 (5). Seidah et al. (5), using northern blot analysis in different cell lines, found that PCSK9 is mainly expressed in the liver of humans as well as in the small intestine, liver, and pancreatic islets of rodents. PCSK9 consists of three structural domains: the prodomain, the catalytic subunit, and the C-terminal Cys/His-rich domain (CHRD) (6, 7). PCSK9 maps to the short arm of human chromosome 1p32, implicating it as a possible third gene involved in cholesterol metabolism after low-density lipoprotein receptor (LDLR) and apolipoprotein B (ApoB) (5, 8, 9). A large number of studies have subsequently provided evidence for this hypothesis. In gene regulation, PCSK9 is regulated by dietary cholesterol and sterol regulatory element binding proteins 1a (SREBP1a) or SREBP2 (10). Statins upregulate the transcription levels of PCSK9 and LDLR (11). Abifadel et al. (12) first revealed that PCSK9 mutations lead to autosomal dominant hypercholesterolemia. Subsequent mechanism studies demonstrated that PCSK9 induces liver LDLR degradation through the lysosomal pathway (13, 14). Besides, human circulating PCSK9 levels are directly correlated with serum low-density lipoprotein cholesterol (LDLc) levels (15, 16). Heterozygote loss-of-function mutations in human PCSK9 are associated with reduced LDLc levels and are accompanied by a significant reduction in the incidence of acute cardiovascular and cerebrovascular events (17–20). In animal studies, *Pcsk9* knockout mice showed a 40-50% decrease in circulating total cholesterol levels, an 80% decrease in LDLc levels, and an increase in hepatic LDLR protein levels (21, 22). In the atherosclerotic mouse model, *Pcsk9* overexpression promotes atherosclerosis, whereas deletion is protective (23, 24). The main research on the PCSK9 mouse model in lipid metabolism are summarized in **Table 1**.

**Table 1:** The main research on the PCSK9 mouse model in lipid metabolism.

Author (Reference)	Year	Consequences
Rashid S (21)	2005	PCSK9 knockout mice showed an increase in liver LDLR protein and a decrease in plasma cholesterol levels. PCSK9 inhibitors may have synergistic effects with statins
Zaid A (22)	2008	PCSK9 mainly regulates cholesterol homeostasis via LDLR. PCSK9 deficiency causes resistance to liver steatosis
Rousselet E (25)	2011	PCSK9 inhibition failed to generate an apparent neuronal phenotype
Roubtsova A (26)	2011	PCSK9 knockout is associated with upregulation of VLDLR levels in adipose tissue and the accumulation of triglycerides
Denis M (23)	2012	PCSK9 absence prevents atherosclerosis, while PCSK9 overexpression promotes it
Demers A (27)	2015	PCSK9 induces CD36 degradation and affects fatty acid uptake and triglyceride metabolism in liver tissue
Roubtsova A (28)	2015	The distribution of LDLR and VLDLR caused by PCSK9 deficiency is related to gender and tissue specificity
Tang ZH (29)	2017	PCSK9 silencing inhibits atherosclerosis without changing plasma cholesterol levels by reducing vascular inflammation and blocking the TLR4/NF-κB signaling pathway

**Figure 1:**
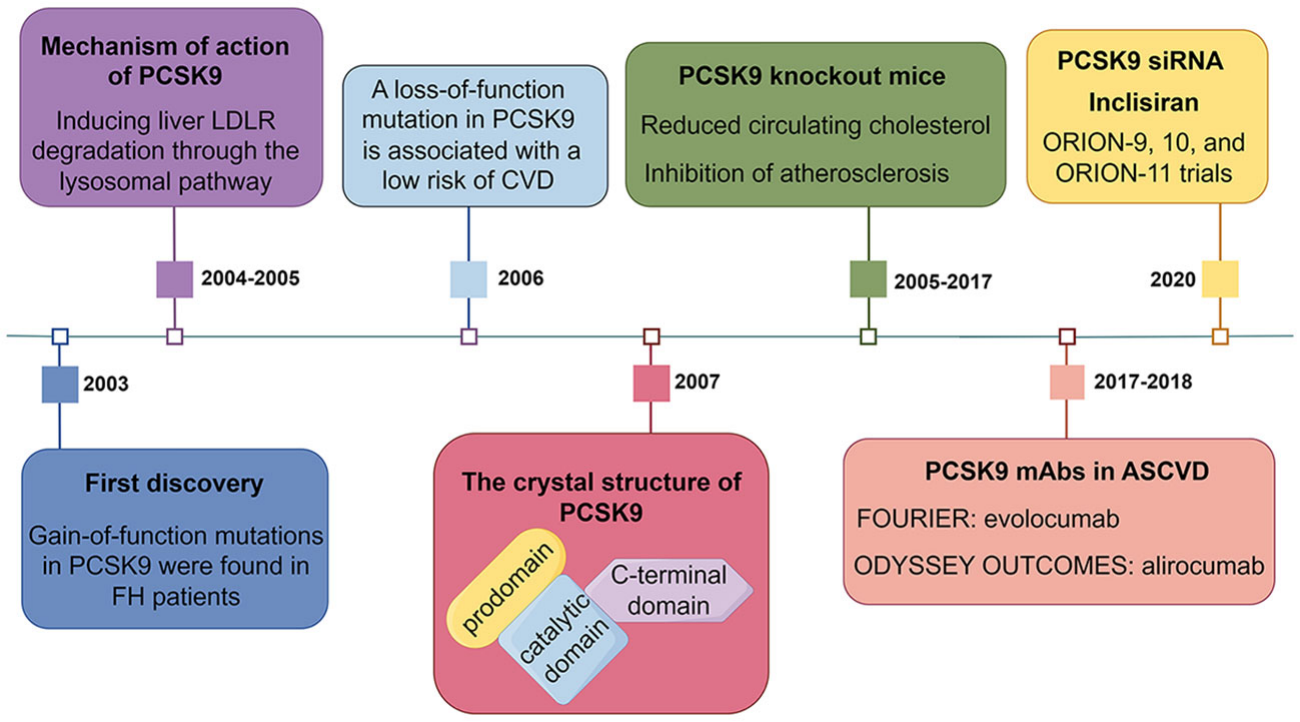
A timeline of significant findings on PCSK9 in lipid metabolism. LDLR, low-density lipoprotein receptor; FH, familial hypercholesterolemia; CVD, cardiovascular disease; mAbs, monoclonal antibodies; ASCVD, atherosclerotic cardiovascular disease; siRNA, small interfering RNA.

Currently, the most widely used PCSK9 inhibitors in the clinic are PCSK9 monoclonal antibodies (mAbs) (evolocumab and alirocumab), which inhibit the binding of circulating PCSK9 to the LDLR. Alirocumab monotherapy or in combination with other lipid-lowering drugs reduces LDLc levels by 40 - 70%, although statins upregulate PCSK9 expression through activation of SREBP2 (11, 30–32). FOURIER and ODYSSEY are two large randomized controlled studies aimed at evaluating the role of PCSK9 inhibitors in the secondary prevention of atherosclerotic cardiovascular disease (ASCVD) (33, 34). Evolocumab and alirocumab cumulatively reduced the relative risk of major cardiovascular events by 15% in patients at 3 and 4 years of follow-up, respectively (33, 34). Studies have shown that PCSK9 mAb is well suited for normal and heterozygote familial hypercholesterolemia (HeFH) patients due to its ability to prevent LDLR degradation (35). Another marketed PCSK9 inhibitor is the PCSK9 small interfering RNA (siRNA) (inclisiran), which targets the liver and prevents PCSK9 translation (36). Unlike biweekly or monthly injections of PCSK9 mAb, injections of PCSK9 siRNA twice a year still reduced LDLc levels by 50 - 60% (37). A timeline of significant findings on PCSK9 in lipid metabolism is presented in **Figure 1**.

Undoubtedly, PCSK9 inhibitors are brilliant stars in the field of lipid metabolism. In addition, the role of PCSK9 in sepsis, vascular inflammation, and tumor immunity is also receiving increasing attention (38). The number of research publications on PCSK9 inhibitors shows an increasing trend year by year. For the field of PCSK9 inhibitors, however, there are no relevant studies with bibliometric analysis. Therefore, we review the related literature on PCSK9 inhibitors and analyze the related data using the bibliometric software CiteSpace, aiming to intuitively present the hotspots and evolution trends of PCSK9 inhibitor-related research and provide new ideas and references for future research.

## 2 Materials and methods

### 2.1 Literature search and data collection

**Figure 2:**
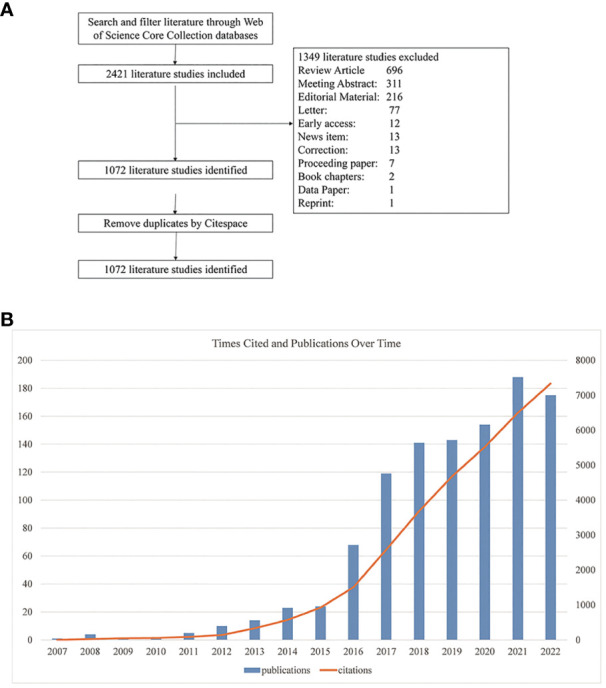
**(A)** Flowchart of the literature search and selection process. **(B)** Global trend of annual publications and citations related to PCSK9 inhibitors from 2007 to 2022.

We implemented a systematic literature search through the Web of Science Core Collection (WoSCC) database using the following strategy: TS = (“proprotein convertase subtilisin/kexin type 9 inhibitor*” OR “pcsk9 inhibitor*” OR “proprotein convertase subtilisin/kexin type 9 inhibition*” OR “pcsk9 inhibition”) AND LA=(English), from 1999 to December 31, 2022. The results showed that the first article on PCSK9 inhibitors was published in 2007. A total of 2421 records were retrieved. We then excluded 1349 literature studies on review articles, meeting abstracts, editorial material, letters, early access, news items, corrections, proceeding papers, book chapters, data papers, and reprints. The final search yielded 1072 papers. The procedure for searching was presented in **Figure 2A**.

### 2.2 Data analysis

The retrieved literature data were exported and then imported into CiteSpace software (version 6.2. R2) for further analysis and processing (39). Information extracted from each study included authors, keywords, affiliations, countries and regions, publication years, publication titles, and funding agencies. The visual maps drawn by CiteSpace consist of nodes and connections. Nodes typically denote specific items like authors, nations, or institutions, and their size and color indicate the amount and type of items accordingly. The number of connections between nodes shows how frequently items are co-cited or collaborated upon. The higher a node’s centrality, the more impact it has in the field, as centrality is a measure of importance in a relationship network. We also use Microsoft Excel (version 2019) and Graphpad software (version 9.4.1) for drawings.

## 3 Results

### 3.1 The global overview of publication outputs

The number of publications reveals the trend and rate of research development in a certain field. As shown in **Figure 2B**, the annual volume of literature in this field was less than 30 before 2016; it grew rapidly from 2016 onward, with 175 papers published by 2022. Similar to the number of publications, the citation volume of literature has also grown rapidly year by year. It’s worth noting that the number of articles published in 2022 was lower than in 2021; this could be because of the coronavirus disease 2019 (COVID-19) epidemic, which temporarily shifted the focus of scientific research. As shown in **Table 2**, the main research areas include cardiac cardiovascular systems (422, 39.37%), pharmacology pharmacy (226, 21.08%), and peripheral vascular disease (132, 12.31%), etc. For journal ranking, most papers related to PCSK9 inhibitors were published in the following journals: *Journal of Clinical Lipidology* (66, 6.16%), *Atherosclerosis* (45, 4.2%), *Journal of the American College of Cardiology* (31, 2.89%), *Circulation* (25, 2.33%), and *Journal of the American Heart Association* (25, 2.33%). In terms of funding, Amgen grants supported the most research (200, 18.66%), followed by two major pharmaceuticals, Regeneron (126, 11.75%) and Sanofi (103, 9.61%).

**Table 2:** Top five or ten based on the number of documents (2007–2022).

Field		Count	% of 1072	Centrality
Research areas	Cardiac cardiovascular systems	422	39.37	
	Pharmacology pharmacy	226	21.08	
	Peripheral vascular disease	132	12.31	
	Medicine general internal	113	10.54	
	Biochemistry molecular biology	74	6.90	
Journals	Journal of Clinical Lipidology	66	6.16	
	Atherosclerosis	45	4.20	
	Journal of the American College of Cardiology	31	2.89	
	Circulation	25	2.33	
	Journal of the American Heart Association	25	2.33	
Funding Agencies	Amgen	200	18.66	
	Regeneron	126	11.75	
	Sanofi	103	9.61	
	United States Department Of Health Human Services	79	7.37	
	National Institutes Of Health Nih Usa	76	7.09	
Countries	United States	507	47.29	0.05
	England	163	15.21	0.03
	Canada	133	12.41	0.01
	Italy	131	12.22	0.02
	France	128	11.94	0.06
	Germany	120	11.19	0.05
	China	109	10.17	0.00
	Australia	103	9.61	0.04
	Netherlands	102	9.51	0.01
	Spain	68	6.34	0.03

### 3.2 Analysis of countries and institutions

**Figure 3:**
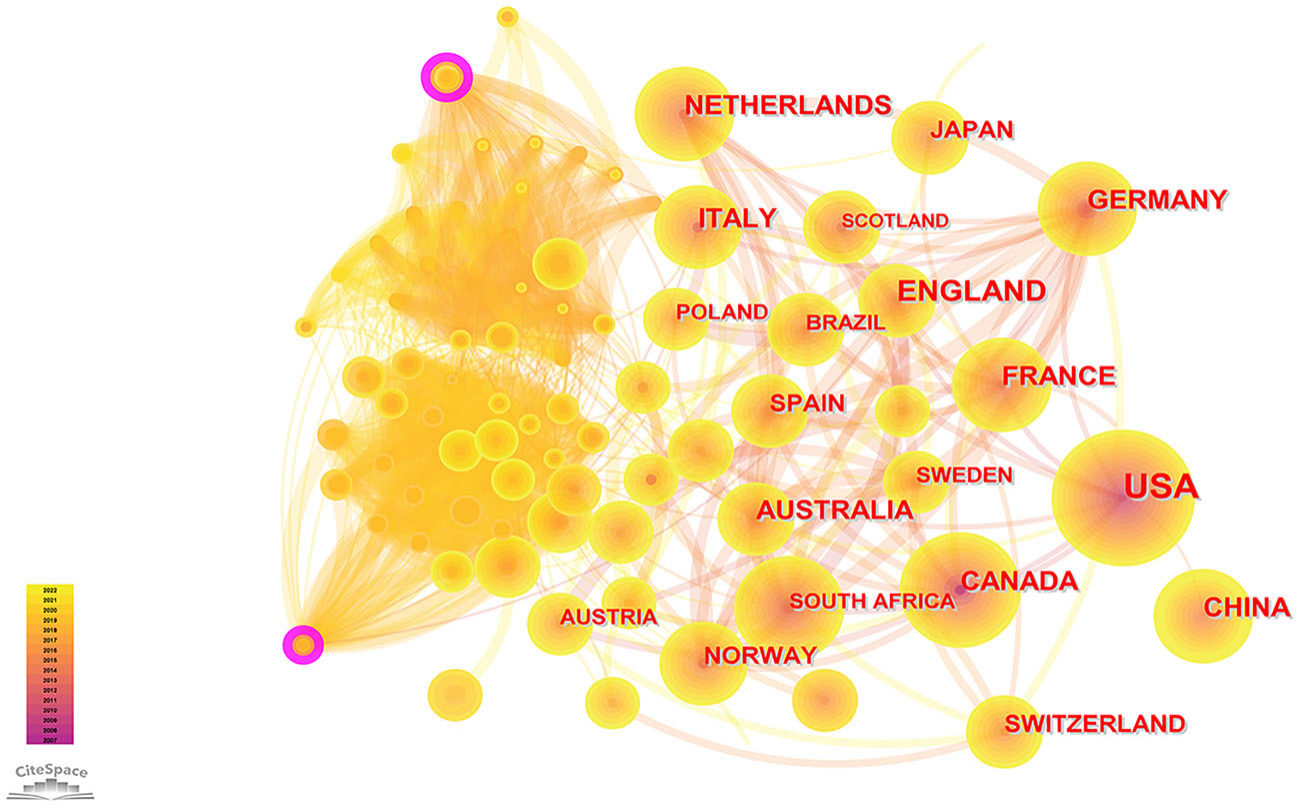
Visual maps of publications over countries studying PCSK9 inhibitors. Each node represents a country, and node size indicates the number of publications. The connection between the nodes represents a co-occurrence relationship. The figure shows the top countries in terms of the number of published articles. The USA ranks first, and the other countries are England, Canada, Italy, and France. The lines between the nodes represent the cooperation between different countries.

**Figure 4:**
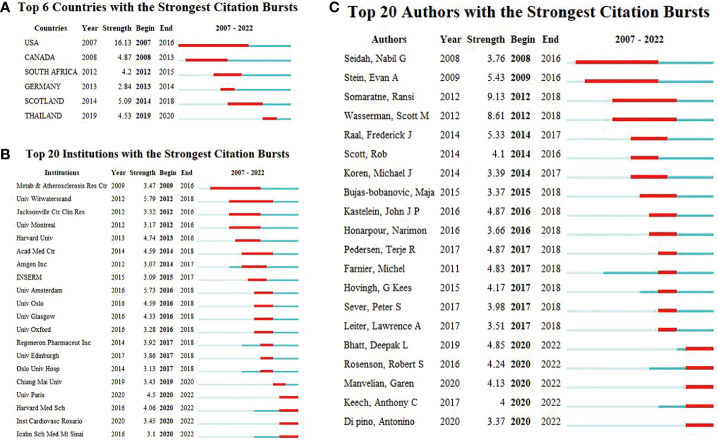
**(A)** Top six countries. **(B)** Top 20 institutions, and **(C)** Top 20 authors with the strongest citation bursts (2007–2022). Burst refers to a sudden increase in the number of citations in a certain period, suggesting an increased research in such a field. The red bands indicate the duration of burst.

**Figure 5:**
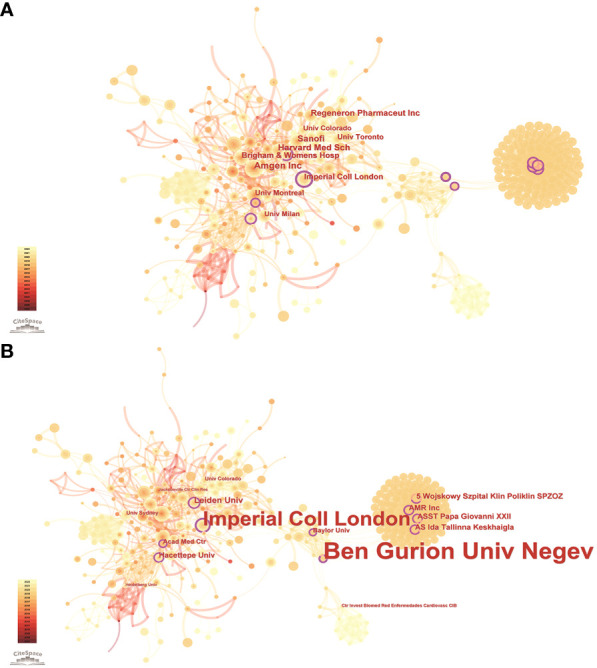
Publications over institutions. The node size represents the number of articles. The larger the node, the greater the number of articles. **(A)** The highest number of publications is from Amgen Inc, Harvard Med Sch, Sanofi, Regeneron Pharmaceut Inc, Brigham & Womens Hosp, Imperial Coll London, Univ Montreal, Univ Toronto, Univ Milan, Univ Colorado. **(B)** The highest centrality institutions are Ben Gurion Univ Negev, Imperial Coll London, Leiden Univ, Hacettepe Univ, ASST Papa Giovanni XXII, AMR Inc, Wojskowy Szpital Klin Poliklin SPZOZ, AS Ida Tallinna Keskhaigla, Acad Med Ctr, Baylor Univ. The lines between the nodes represent the cooperation between the different institutions.

According to the WoSCC database, among the 89 countries or regions involved in the PCSK9 inhibitors field, the country with the highest volume of publications is the United States (507, 47.29%), followed by England (163, 15.21%), Canada (133, 12.41%), Italy (131, 12.22%), and France (128, 11.94%) (**Table 2**). Through CiteSpace screening, **Figure 3** visualizes the outputs and links across countries. Among the top 10 countries in output ranking (**Table 2**), France, the United States, and Germany have the highest centrality, denoting their important role in the field of PCSK9 inhibitors. Further, from 2007 through 2022, the publications with the strongest bursts of citations in countries were analyzed. In **Figure 4**, the blue segment represents the timeline, while the red line represents the citation burst duration. The top 6 countries for the strongest citation bursts (**Figure 4A**) include the United States of America (USA) (2007–2016), Canada (2008–2013), South Africa (2012–2015), Germany (2013–2014), Scotland (2014–2018), and Thailand (2019–2020). Among these countries, the USA (16.13) showed the highest burst strength and longest duration. According to the WoSCC database, the top 5 institutions for article output in the field of PCSK9 inhibitor research (**Table 3**) are Harvard University (128, 11.94%), Amgen Inc. (114, 10.63%), Brigham & Women’s Hospital (107, 9.98%), Harvard Medical School (94, 8.77%), and Imperial College London (83, 7.74%). In the collaboration network (**Figure 5A**) and centrality ranking (**Figure 5B**) drawn by CiteSpace, the three institutions with the highest centrality are Ben Gurion Univ Negev (0.4), Imperial College London (0.35), and Leiden Univ (0.14), respectively, indicating their important position in the field, although some of them have relatively fewer publications. In addition, we conducted an analysis of citation bursts from various institutions over the past fifteen years (**Figure 4B**). Among these, the top-ranked institutions for citation bursts include: Univ Witwatersrand (5.79), Univ Amsterdam (5.73), and Harvard Univ (4.74), representing the highest research heat in the early years. In recent years, the Univ Paris, Harvard Med Sch, Icahn Sch Med Mt Sinai, and Inst Cardiovasc Rosario have also shown high enthusiasm for research.

**Table 3:** Top 5 affiliations and centrality ranking in PCSK9 inhibitors (2007–2022).

Rank	Affiliations	Count	% of 1072	Affiliations	Centrality	Count
1	Harvard University	128	11.94	Ben Gurion Univ Negev	0.4	8
2	Amgen	114	10.63	Imperial Coll London	0.35	51
3	Brigham & Women’s Hospital	107	9.98	Leiden Univ	0.14	29
4	Harvard Medical School	94	8.77	Hacettepe Univ	0.13	12
5	Imperial College London	83	7.74	ASST Papa Giovanni XXII	0.13	5

### 3.3 Analysis of authors

According to the WoSCC database, **Table 4** enumerates the top 6 authors with the highest number of publications in the field of PCSK9 inhibitors: Sabatine MS (51, 4.76%), Giugliano RP (46, 4.29%), Wasserman SM (38, 3.54%), Mach F (31, 2.89%), Jukema JW (29, 2.71%), and Somaratne R (29, 2.71%). In the ranking of centrality (**Table 4**), the top-ranked authors included: Wasserman SM (0.07), Cannon CP (0.06), Catapano AL (0.06), Blom DJ (0.06), Banach M (0.05), and Ballantyne CM (0.05). **Figure 4C** presents a list of the top 20 authors for citation bursts over the past 15 years. The two authors with the highest burst strength are Somaratne R (9.13) and Wasserman SM (8.61), who are much higher than the other authors.

**Table 4:** Top 6 authors and centrality ranking in PCSK9 inhibitors (2007–2022).

Rank	Author	Count	% of 1072	Author	Centrality	Count	Year
1	Sabatine MS	51	4.76	Wasserman SM	0.07	35	2012
2	Giugliano RP	46	4.29	Cannon CP	0.06	17	2015
3	Wasserman SM	38	3.54	Catapano AL	0.06	12	2017
4	Mach F	31	2.89	Blom DJ	0.06	7	2015
5	Jukema JW	29	2.71	Banach M	0.05	17	2012
6	Somaratne R	29	2.71	Ballantyne CM	0.05	14	2015

### 3.4 Highly cited references

**Figure 6:**
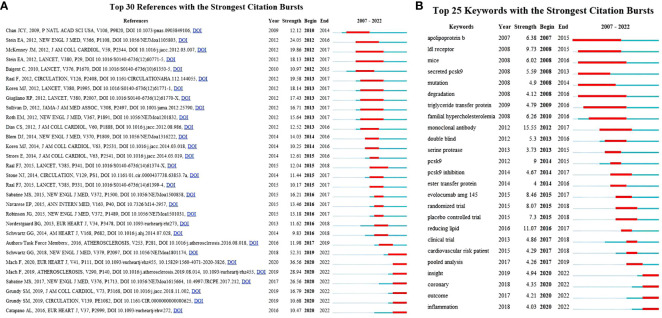
**(A)** The top 30 references with the strongest citation bursts. The red segment represents the begin and end years of the burst duration. **(B)** Top 25 keywords with the strongest citation bursts (2007–2022). Burst refers to a sudden increase in the number of citations in a certain period, suggesting an increased research in such a field. The red bands indicate the duration of burst.

Highly cited references indicate that the academic quality and impact of the paper are widely recognized. Highly cited references can reflect hotspots in the field and are the focus of researchers. **Table 5** shows the 10 most frequently cited literatures in terms of PCSK9 inhibitors. More than half of these 10 literatures were about clinical trials of PCSK9 mAbs, and almost all were published in the *New England Journal of Medicine*. The first-, fourth-, seventh-, and eighth-ranked literature examined the effect of evolocumab on LDLc and the risk of cardiovascular events, whereas the second- and third-ranked literature examined the effect of alirocumab on LDLc and the risk of cardiovascular events. The top 30 references, based on the citation bursts with the highest strength, are shown in **Figure 6A**. The article “Alirocumab and Cardiovascular Outcomes after Acute Coronary Syndrome”, published in 2018, wins the strongest citation burst. This article has a burst intensity of 52.31, and its heat might continue.

**Table 5:** Top 10 high-cited literatures related to PCSK9 inhibitors.

Rank	Title	Authors	Journal	Year	Citation
1	Evolocumab and Clinical Outcomes in Patients with Cardiovascular Disease	Sabatine MS	New England Journal of Medicine	2017	439
2	Alirocumab and Cardiovascular Outcomes after Acute Coronary Syndrome	Schwartz GG	New England Journal of Medicine	2018	224
3	Efficacy and safety of alirocumab in reducing lipids and cardiovascular events	Robinson JG	New England Journal of Medicine	2015	201
4	Efficacy and safety of evolocumab in reducing lipids and cardiovascular events	Sabatine MS	New England Journal of Medicine	2015	150
5	Ezetimibe Added to Statin Therapy after Acute Coronary Syndromes	Connon CP	New England Journal of Medicine	2015	149
6	2019 ESC/EAS Guidelines for the management of dyslipidaemias: lipid modification to reduce cardiovascular risk	Mach F	European Heart Journal	2020	97
7	PCSK9 inhibition with evolocumab (AMG 145) in heterozygous familial hypercholesterolaemia (RUTHERFORD-2): a randomised, double-blind, placebo-controlled trial	Raal FJ	Lancet	2015	93
8	A 52-week placebo-controlled trial of evolocumab in hyperlipidemia	Blom DJ	New England Journal of Medicine	2014	90
9	Low-density lipoproteins cause atherosclerotic cardiovascular disease. 1. Evidence from genetic, epidemiologic, and clinical studies. A consensus statement from the European Atherosclerosis Society Consensus Panel	Ference BA	European Heart Journal	2017	85
10	2013 ACC/AHA guideline on the treatment of blood cholesterol to reduce atherosclerotic cardiovascular risk in adults: a report of the American College of Cardiology/American Heart Association Task Force on Practice Guidelines	Stone NJ	Circulation	2014	81

### 3.5 Highly cited journals

**Figure 7:**
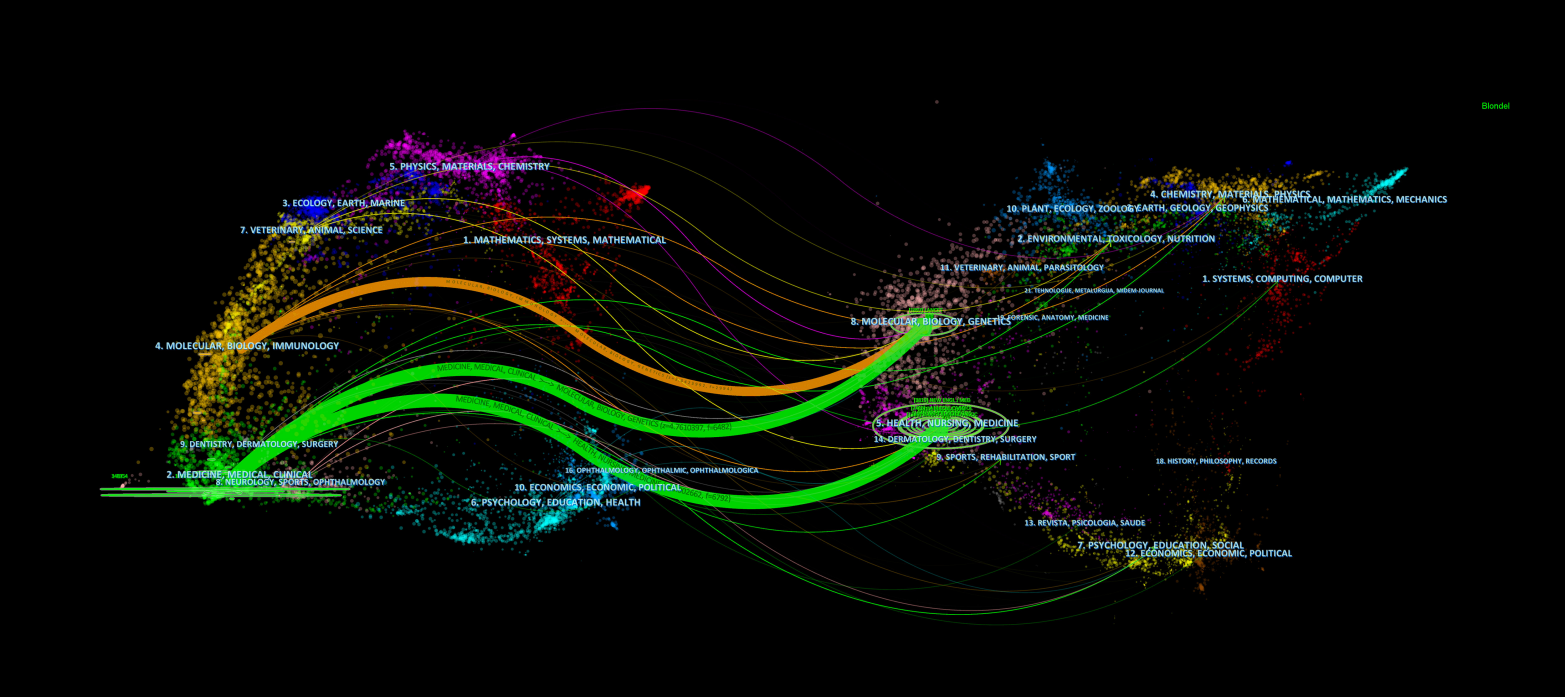
The dual map overlay of journals contributed to publications on the application of PCSK9 inhibitors from 2007 to 2022. All the orange paths showed articles in the research fields of molecular/biology/immunology are more likely to cite articles in the field of molecular, biology, genetics. While the green path showed articles in medicine/medical/clinical fieds are divided into two parts, one is health/nursing/medicine, the other is molecular/biology/genetics.

The top 10 journals according to the number of citations are shown in **Table 6**. The *New England Journal of Medicine* (864) was the most cited journal, followed by *Circulation* (747), the *Journal of the American College of Cardiology* (739), the *European Heart Journal* (674), the *Lancet* (632), the *Atherosclerosis* (624), the *Jama-Journal of the American Medical Association* (565), the *Journal of Clinical Lipidology* (442), the *American Journal of Cardiology* (367), and the *Journal of Lipid Research* (345). The 2021 impact factor for these journals ranged from 3.133 to 202.731, with 60% of journals having an impact factor exceeding 20. Through journal citation reports (JCR) partition analysis, Q1 accounts for 90%. In addition, we also constructed a dual map overlay of journals on PCSK9 inhibitors (**Figure 7**) to describe the subject distribution of journals. The left part of the figure shows the citing journals, while the right part shows the cited journals. Three main citation pathways are shown in this figure. The two green citation lines indicate that research in Molecular, Biology, Genetics journals and Health, Nursing, Medicine journals is frequently cited by Medicine, Medical, Clinical journals. The orange citation line indicates that research in Molecular, Biology, Genetics journals is frequently cited by Molecular, Biology, Immunology journals.

**Table 6:** Top 10 cited journals from 2007 to 2022.

Rank	Journal	Count	Impact Factor	Journal Citation Reports
1	NEW ENGL J MED	864	176.082	Q1
2	CIRCULATION	747	39.922	Q1
3	J AM COLL CARDIOL	739	27.206	Q1
4	EUR HEART J	674	35.855	Q1
5	LANCET	632	202.731	Q1
6	ATHEROSCLEROSIS	624	6.851	Q1
7	JAMA-J AM MED ASSOC	565	157.375	Q1
8	J CLIN LIPIDOL	442	5.365	Q1
9	AM J CARDIOL	367	3.133	Q3
10	J LIPID RES	345	6.676	Q1

### 3.6 Analysis of keywords

**Figure 8:**
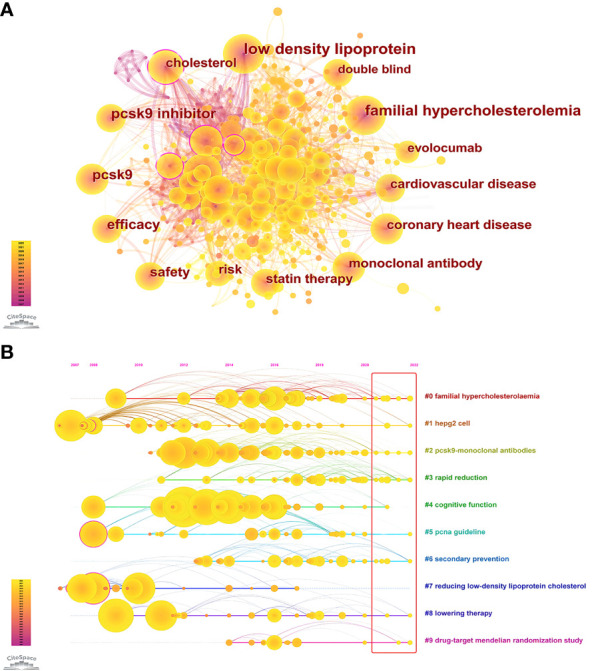
**(A)** Co-occurrence Analysis of keywords. Each node represents a keyword, and node size indicates the number of occurrences. The connection between the nodes represents a co-occurrence relationship. The top ten co-occurrence keywords are “low density lipoprotein” (391), “familial hypercholesterolemia” (305), “pcsk9 inhibitor” (265), “pcsk9” (230), “efficacy” (221), “safety” (187), “risk” (164), “cholesterol” (159), “cardiovascular disease” (158), and “monoclonal antibody” (149). **(B)** Circles indicated keywords; the size of the diameter of the circle was closely related to the number of occurrences; those with purple circles stated the existence of significant turning points in scientific knowledge, i.e., higher centrality; the purple axis indicated 2007-2022; yellow circles still in the red rectangle indicated the occurrence of novel related keywords.

The keywords are the core and main points of a paper and can reflect the hotspots and fronts of a certain field. **Table 7** and **Figure 8A** show the co-occurrence analysis of keywords. The keywords with a high frequency of co-occurrence include “low density lipoprotein” (391), “familial hypercholesterolemia” (305), “pcsk9 inhibitor” (265), “pcsk9” (230), “efficacy” (221), “safety” (187), “risk” (164), “cholesterol” (159), “cardiovascular disease” (158), and “monoclonal antibody” (149). The keyword centrality ranking (**Table 7**) shows that “apolipoprotein b” (0.13), “atorvastatin” (0.11), “expression” (0.11), “cholesterol” (0.1), and “ldl receptor” (0.09) have higher centrality than other keywords, indicating that these keywords are the core of the PCSK9 inhibitors field. We further conducted the keyword cluster timeline using CiteSpace. Keyword clusters refer to groups consisting of keywords with similar research topics. A total of ten clusters are generated as shown in **Figure 8B**: “familial hypercholesterolaemia”, “hepg2 cell”, “pcsk9-monoclonal antibodies”, “rapid reduction”, “cognitive function”, “pcna guideline”, “secondary prevention”, “reducing low-density lipoprotein cholesterol”, “lowering therapy”, and “drug-target mendelian randomization study”. Circles denoted keywords in the keyword clustering timeline, and the number of occurrences was closely correlated with the circle’s diameter; the purple axis indicated 2007-2022. Meanwhile, we present the top 25 keywords in the field of PCSK9 inhibitors that have had the strongest citation bursts within the past 15 years (**Figure 6B**). Notably, “monoclonal antibody” (15.55) has the strongest citation bursts, followed by “reducing lipid” (11.07), “ldl receptor” (9.73), “pcsk9” (9), “evolocumab amg 145” (8.46), and “randomized trial” (8.07). As can be seen from **Figure 6B**, “apolipoprotein b”, “ldl receptor”, “mice”, “secreted pcsk9”, “mutation”, “degradation”, “triglyceride transfer protein”, and “familial hypercholesterolemia” were the earliest keywords in the field of PCSK9 inhibitors and were therefore the subjects of interest to researchers in the early years. Moreover, “insight”, “coronary”, “outcome”, and “inflammation” are at the forefront of the current PCSK9 inhibitor research field and are in a period of explosion.

**Table 7:** Keywords co-occurrence frequency and centrality ranking (Top 35, 2007–2022).

Keywords	Count	Centrality	Year	Keywords	Centrality	Count	Year
low density lipoprotein	391	0.04	2008	apolipoprotein b	0.13	27	2007
familial hypercholesterolemia	305	0.02	2008	atorvastatin	0.11	70	2011
pcsk9 inhibitor	265	0.04	2009	expression	0.11	58	2008
pcsk9	230	0.01	2012	cholesterol	0.1	159	2007
efficacy	221	0.01	2012	ldl receptor	0.09	65	2008
safety	187	0.01	2013	cardiovascular disease	0.08	158	2011
risk	164	0.07	2014	risk	0.07	164	2014
cholesterol	159	0.1	2007	secondary prevention	0.07	34	2009
cardiovascular disease	158	0.08	2011	risk factor	0.07	29	2014
monoclonal antibody	149	0.03	2012	degradation	0.07	21	2008
coronary heart disease	143	0.06	2008				
statin therapy	141	0.03	2013				
disorder	119	0.02	2012				
evolocumab	116	0.02	2015				
therapy	107	0.02	2014				
double blind	106	0.05	2012				
reducing lipid	94	0.01	2016				
meta analysis	91	0.01	2016				
management	79	0.02	2016				
hypercholesterolemia	78	0.06	2008				
pcsk9 inhibition	75	0.04	2014				
atorvastatin	70	0.11	2011				
statin	68	0.04	2010				
alirocumab	67	0	2016				
ezetimibe	67	0.02	2010				
ldl receptor	65	0.09	2008				
myocardial infarction	61	0.06	2009				
expression	58	0.11	2008				
association	58	0.06	2014				
lipid-lowering therapy	55	0.01	2016				
cardiovascular risk	50	0.02	2013				
mutation	49	0.05	2008				
atherosclerosis	46	0.02	2016				
amg 145	45	0.05	2013				
guideline	43	0.02	2012				

## 4 Discussion

### 4.1 General tendency of research

In this study, the literature related to PCSK9 inhibitors was analyzed using CiteSpace quantitative analysis software. Publications, nations, organizations, writers, keywords, literature, journals, and other objects were all subjected to a quantitative evaluation. From the number of publications (**Figure 2B**), very few PCSK9 inhibitor-related articles existed before 2012. The possible reason is that researchers have focused mainly on animal models and the mechanism of the decrease in LDLc caused by PCSK9 deficiency (40–45). Since 2012, with the continuous development of clinical trials of PCSK9 mAbs (46–50), the output of papers in this field has also been increasing year by year. Given the large number of clinical trials of PCSK9 mAbs demonstrating efficacy and safety for lipid lowering (51–53), the US Food and Drug Administration and the European Medicines Agency approved evolocumab and alirocumab, two PCSK9 mAbs, in 2015 for LDLc lowering, particularly in patients with familial hypercholesterolemia (FH). Thus, the number of publications has grown rapidly since 2015. In 2019, with the release of the results of phase 3 trials of inclisiran (54), a PCSK9 siRNA, this field is gaining more attention, even though the paper output is tempered by the outbreak of COVID-19.

As can also be seen in **Table 5**, multiple heavyweight papers were published in 2015, all of which were published in the *New England Journal of Medicine* or the *Lancet*. These articles, including the two most frequently cited ones (Sabatine MS, 2017 and Schwartz GG, 2018), are almost exclusively large clinical trials of PCSK9 mAbs, indicating that clinical trials of PCSK9 inhibitors have received significant attention from researchers and also demonstrating their strong interest and recognition in the clinical application of PCSK9 inhibitors. In addition, two of the top 10 articles were about dyslipidemia management guidelines, demonstrating the important role of guidelines in guiding clinical and scientific research work. As we can see from **Table 2**, the USA, England, Canada, Italy, and France are the five countries with the largest number of published articles. Notably, the number of publications for American academics was almost the sum of the number of publications from the other four countries. The top five institutions by number of publications include Harvard University, Amgen Inc., Brigham & Women’s Hospital, Harvard Medical School, and Imperial College London, with four institutions located in the United States and one in England. In a similar vein, four of the top five funding organizations are American, and one is French. Consistent with the countries citation bursts intensity (**Figure 4A**), the United States undoubtedly holds a leading position in the field of PCSK9 inhibitors due to excellent research institutions and sufficient financial support. In the cooperation network of countries (**Figure 3**), there is close cooperation and communication between developed countries, such as France, the United States, Germany, and so on. It is worth noting that although China is the only Asian country in the top 10 ranking for the number of publications (**Table 2**), there is little cooperation between China and other countries. And there were not many high-quality studies published in top journals in China. Further analysis of countries bursts (**Figure 4A**) show that South Africa and Thailand exhibit research enthusiasm in this area, in addition to developed countries such as Europe and the United States. The relatively lagging biomedical industry may be the reason why developing countries such as China have published fewer papers in this field. In addition, the use of PCSK9 inhibitors is particularly constrained in underdeveloped countries by the high expenditures associated with research and development (R&D) and clinical trials. China has abundant biological resources and a huge market demand. With the government’s support, gradually increased R&D input, and enhanced cooperation and communication with other countries, it is believed that China will also achieve high-quality research in this field in the future. Four American authors, one Swiss author, and one Dutch author make up the top-ranked authors for the number of published articles (**Table 4**). Sabatine MS and Giugliano RP work at Brigham and Women’s Hospital. Wasserman SM and Somaratne R are both employed by Amgen. Mach F works at the University of Geneva. Jukema JW is from Leiden University Medical Center. It is worth mentioning that two professors, Sabatine MS and Giugliano RP, who have been long dedicated to the research of PCSK9 inhibitors in clinical trials since 2012 and have continuously presented high-quality papers to date, have made important contributions to the clinical applications of PCSK9 inhibitors. The top-ranked author for centrality is Wasserman SM from Amgen (**Table 4**), who has also been involved in a large number of clinical trials of PCSK9 inhibitors, undoubtedly having a profound impact on the development of this field. Overall, our results demonstrate consistency among high-yield countries, institutions, authors, and funding agencies. Developed countries such as the United States and Western European countries have been leading the way in the study of PCSK9 inhibitors. In addition, developing countries such as China should enhance their exchanges and cooperation with other countries to advance this field.

Journal and cited journal ranking analysis helps researchers select the most appropriate journal for their manuscript (55, 56). **Table 2** displays the relative number of PCSK9 inhibitor-related publications published in many medical journals. The *Journal of Clinical Lipidology* (Q1, IF=5.365) leads the pack, followed by *Atherosclerosis* (Q1, IF=6.847), the *Journal of the American College of Cardiology* (Q1, IF=27.203), *Circulation* (Q1, IF=39.918), and the *Journal of the American Heart Association* (Q2, IF=6.106). This result suggests that high-quality journals favor relevant studies on PCSK9 inhibitors. Of the ranking of cited journals (**Table 6**), 90% are high impact Q1 international journals. Among these journals, the *New England Journal of Medicine* and *Journal of the American College of Cardiology* focus more on the related clinical studies of PCSK9 inhibitors, while the *Journal of Lipid Research* et al. emphasize the early basic research in the PCSK9 field. This means that the field of PCSK9 inhibitors is currently relatively mature, either in basic or clinical studies. Moreover, the dual journal map overlay (**Figure 7**) reveals that “molecular, biology, genetics” are frequently cited in “medicine, medical, clinical” demonstrating that current PCSK9 inhibitor research is increasingly concentrated on clinical translation.

### 4.2 Research focuses and frontiers

Keywords help understand the focus and evolution of specific research areas. Based on the keyword co-occurrence analysis, keyword timeline, and citation bursts, we believe that the research focuses of PCSK9 inhibitors mainly include the following aspects: lipid-lowering mechanisms of PCSK9 inhibitors, PCSK9 mAbs and related clinical trials.

#### 4.2.1 Lipid-lowering mechanisms of PCSK9 inhibitors

Keywords before 2012 included: apolipoprotein B, LDL receptor, mice, secreted pcsk9, degradation, and so on. These keywords appear to correspond to basic research on PCSK9, suggesting that earlier research focused on the mechanisms of lipid lowering by PCSK9 inhibitors. PCSK9 is the third gene related to cholesterol metabolism after ApoB and LDLR; its mechanism of action was first revealed in 2004 by Maxwell and Breslow et al. (13, 14). They found that PCSK9 reduces LDLR protein levels by inducing LDLR degradation in HepG2 cells. Further studies revealed that PCSK9 may induce LDLR degradation through both circulating and intracellular pathways. Through its catalytic subunit, PCSK9 secreted by the liver binds the EGF-A domain of the LDLR and directs it to endosomes/lysosomes for degradation (45, 57, 58). In addition, several lines of evidence support the relatively rapid intracellular pathway of LDLR degradation by PCSK9 through the *trans*-Golgi to lysosomes (59). For example, autosomal recessive hypercholesterolemia protein (ARH), an adaptor protein, is required for PCSK9-LDLR complex internalization by hepatocytes (60, 61). However, overexpression of *Pcsk9* in *Arh* ^-/-^ mice still reduced hepatic LDLR protein levels (62). Blocking the clathrin light chain, which is responsible for intracellular trafficking from the *trans*-Golgi network to lysosomes, increased LDLR levels within HepG2 cells in a PCSK9 dependent manner without affecting the ability of circulating PCSK9 to enhance LDLR degradation (59). Notably, both intracellular and extracellular LDLR degradation pathways of PCSK9 require the presence of the CHRD, suggesting that this domain is a determinant of LDLR degradation by PCSK9 (63–65). However, the absence of the CHRD domain does not affect the binding of PCSK9 to the LDLR, although it impedes PCSK9-LDLR trafficking to lysosomes (65, 66). On the other hand, mutations in the cytoplasmic domain of LDLR did not affect its degradation by PCSK9 (67). These results indicate that an unknown protein is required for PCSK9 to degrade LDLR. Recent studies have proposed that cyclase-associated protein 1 (CAP1) may be a candidate protein that binds to the M1 and M3 domains of the CHRD and enhances the degradation of PCSK9-LDLR in lysosomes (68).

#### 4.2.2 PCSK9 mAbs and related clinical trials

Keywords from 2012 to 2017 include “monoclonal antibody”, “double blind”, “PCSK9 inhibitor”, “randomized trial”, “evolocumab amg 145”, etc., indicating a shift in researchers’ focus from basic to clinical research, with a focus on PCSK9 monoclonal antibodies and randomized controlled trials (RCTs). Anti-PCSK9 neutralizing antibody (mAb1) was first generated in 2009 by Chan et al., who showed that a single injection of mAb1 resulted in an 80% reduction in circulating LDLc levels in cynomolgus monkeys (41). This exciting discovery lays the foundation for the development of PCSK9 mAbs. Subsequently, since 2012, clinical trials of PCSK9 mAbs have sprouted like mushrooms. In a phase 1 trial, REGN727/SAR236553 (Alirocumab), a PCSK9 mAb developed by Regeneron in collaboration with Sanofi, significantly reduced LDLc levels in the healthy population and in heterozygous hypercholesterolemic patients receiving statin therapy. For the latter, it could be reduced by up to 61% (69). Similar results were observed in a clinical trial of AMG145 (Evolocumab), a PCSK9 mAb developed by Amgen (50). All studies have shown that PCSK9 mAbs (evolocumab or alirocumab) with or without statin therapy significantly reduce LDLc in patients with hypercholesterolemia and heterozygous familial hypercholesterolemia (31, 32, 49, 70). In patients with statin intolerance due to muscle related side effects, the results of the GAUSS study showed that evolocumab similarly reduced LDLc levels (48, 71, 72). Notably, individual responses to the PCSK9 inhibitor evolocumab vary widely among patients with homozygous familial hypercholesterolemia (HoFH). Evolocumab did not lower LDLc in patients with HoFH who completely lacked LDLR (73). Because of the residual LDLR expression and differences seen in most patients with HoFH, the PCSK9 inhibitor evolocumab remains capable of lowering LDLc by 20-30% on statins and ezetimibe basis (73–76). Together, these studies fully demonstrate the lipid-lowering efficacy of PCSK9 mAb. In 2016, GLAGOV, a multicenter randomized controlled study, was the first to evaluate the effect of elovocumab on atherosclerosis (77). Through intravascular ultrasonography (IVUS) imaging measurement, after 78 weeks of treatment with evolocumab on the basis of statins, the percent atheroma volume (PAV) of coronary heart disease patients decreased by 0.95% compared with the baseline (77). In 2017-2018, FOURIER and ODYSSEY OUTCOMES, two large-scale RCTs, respectively, confirmed the lipid-lowering effect and cardiovascular benefits of evolocumab and alirocumab in ASCVD, reducing the relative risk of major cardiovascular endpoint events by 15% (33, 34). The HUYGENS study, published in 2022, assessed the effects of evolocumab on nonculprit vessel plaques in patients with non-ST-segment elevation myocardial infarction based on optical coherence tomography (OCT) (78). The study demonstrated that statin therapy with evolocumab for 50 weeks resulted in an increase in minimum fibrous cap thickness that was approximately 2 times that of placebo, significantly improving plaque vulnerability (78). Similarly, the PACMAN-AMI study evaluated the effectiveness of alirocumab in reversing and stabilizing plaques in patients with acute myocardial infarction (79). The results showed that the reduction in percent atheroma volume and maximum lipid core burden index in the alirocumab group was about twice that of the placebo group (79). The recently released FOURIER-OLE study evaluated the safety and efficacy of long-term use of evolocumab, making it the longest study to date exposed to PCSK9 inhibitors (with a longest follow-up time of up to 8 years) (80). This study further strengthens the position of the PCSK9 mAbs in the secondary prevention of ASCVD patients, advocating for the cardiovascular benefits of early and long-term lipid-lowering therapy. Thus, these clinical trials provide a solid foundation for the evidence-based use of PCSK9 inhibitors in lowering LDLc, reversing atherosclerotic plaques, and reducing cardiovascular events.

#### 4.2.3 PCSK9 inhibitors and inflammation

Based on the keyword bursts, in addition to “coronary” (mentioned above), which is now a hotspot and frontier in the field of PCSK9 inhibitors, inflammation research is likewise at the forefront in this area and is now in an explosive period. In the early years, several observational studies found that plasma PCSK9 concentrations were positively correlated with many inflammatory markers, such as white blood cells, high-sensitivity C-reactive protein (hs-CRP), fibrinogen, etc. (81, 82). Notably, in addition to the liver, small intestine, pancreas, and other organs, PCSK9 is also highly expressed in vascular endothelial cells (EC) and vascular smooth muscle cells (VSMCs) (83). Ding et al. found that PCSK9 in vascular EC and VSMCs is regulated by inflammatory factors such as lectin-like ox-LDL receptor-1 (LOX-1) and tumor necrosis factor-α (TNF-α) (84). As is well known, inflammation is involved in the development and progression of ASCVD. These findings led researchers to focus on the association of PCSK9 with inflammation in atherosclerosis. A clinical study showed that elevated circulating PCSK9 concentrations remained associated with new plaque formation after adjustment for LDLc levels (85). In a basic study, PCSK9 silencing inhibits atherosclerosis without changing plasma cholesterol levels by reducing vascular inflammation and blocking the TLR4/NF-κB signaling pathway (29).In addition, PCSK9 expressed by macrophages promoted atherosclerosis by increasing plaque monocyte infiltration and expression of inflammatory markers independent of circulating cholesterol levels (86). These studies indicate that PCSK9 can directly promote atherosclerotic inflammation, independent of lipid metabolism disorders. Next, the researchers studied the effect of PCSK9 inhibitors on vascular inflammation and atherosclerosis. In animal experiments, Landlinger et al. inhibited circulating PCSK9 by vaccination and found that inhibition of PCSK9 resulted in downregulation of intracellular adhesion molecule-1 (ICAM-1) expression in endothelial cells as well as circulating inflammatory markers (87). In a prospective, observational clinical study, Vlachopoulos et al. assessed arterial inflammation by 18F-fluoro-2-deoxy-D-glucose (FDG) positron emission tomography/computed tomography (PET/CT). They found that 12 months of treatment with evolocumab or alirocumab significantly improved arterial inflammation (88). In addition, several clinical trials have also demonstrated the anti-inflammatory effects of PCSK9 inhibitors (89–91).

COVID-19 is characterized by pathological inflammation and thrombosis (92, 93). High levels of circulating LDLc and lipoprotein (a) [Lp(a)] lead to endothelial dysfunction, accompanied by inflammation and atherosclerosis thrombosis, which are enhanced on the basis of COVID-19, thus aggravating the condition (94–96). Given this, statins have been shown to improve both endothelial dysfunction and prognosis in COVID-19 patients due to their ability to reduce LDLc (97). PCSK9 inhibitors can further reduce the circulating LDLc level when combined with statins and can reduce the serum Lp (a) level by approximately 30% (53), making them a candidate drug for COVID-19. In addition, PCSK9 is directly related to the systemic inflammatory response (98, 99). The anti-inflammatory effects of PCSK9 inhibitors have been widely reported, especially for the interleukin-6 (IL-6)-mediated inflammatory signaling pathway (99–101), which is a driving factor for more severe inflammation in COVID-19 (102). A recent observational study found a significant increase in plasma PCSK9 levels in patients with COVID-19 sepsis compared to those without SARS-CoV-2 infection, suggesting that PCSK9 may be a potential biomarker for COVID-19 (103). PCSK9 inhibitors may inhibit viral infection by reducing viral cholesterol levels, such as dengue virus, according to some previous virus related studies (104, 105). In addition to the improvement of endothelial function related to cholesterol metabolism, PCSK9 inhibitors also benefit from cholesterol-independent mechanisms (104). For example, the PCSK9 inhibitors upregulate the expression of antiviral type 1 interferon in host hepatocytes (106). Therefore, COVID-19 patients with a gain-of-function mutation of *PCSK9* or increased circulating PCSK9 levels (like FH) and type 1 interferon immune deficiency may benefit most from PCSK9 inhibitors (104). The results of a recent multicenter clinical trial showed that the use of evolocumab reduces the 30-day mortality rate or intubation requirement in severe COVID-19 patients by 30%, accompanied by a significant reduction in serum IL-6 levels (101, 107). In addition, patients with more severe systemic inflammation experienced a greater decrease in mortality after using PCSK9 inhibitors (101). This study provides compelling evidence that PCSK9 inhibitors directly control inflammation in COVID-19.

#### 4.2.4 PCSK9 inhibitors and tumors

Cholesterol metabolism is crucial to the growth and development of tumors (108). Given that LDLR provides cholesterol to the liver and other tissues, the upregulation of LDLR in tumors is believed to be associated with cancer development (109). Interestingly, the PCSK9 mAbs did not result in a rise in the incidence of cancer despite increasing the levels of LDLR in the liver and other tissues (33). On the contrary, current research has shown that PCSK9 deficiency inhibits tumor growth (110, 111). Recent studies have shown that tumors evade immune responses by expressing PCSK9 to degrade the major histocompatibility type-I receptor (MHC-I) (112). In addition, PCSK9 inhibition enhances the sensitivity of tumors to immune checkpoint therapy (112). Furthermore, Yuan et al. found that PCSK9 damages the anti-tumor activity of CD8^+^ T cells in the tumor microenvironment by inhibiting LDLR-mediated TCR signals (113). These findings are of great significance for the use of PCSK9 inhibitors in clinical tumor treatment.

The great progress of PCSK9 inhibitors from basic research to clinical conversion over the past decade has largely benefited from their strong reduction in LDLc levels and ASCVD risk without significant side effects, as the keywords “efficacy” and “safety” show in **Table 7**. However, some aspects of clinical practice still need attention. Hyporesponsiveness of some patients to PCSK9 mAbs therapy, for example, is defined as a decrease in LDLc levels of less than 15% (114, 115). One important reason is improper entry and distribution of antibodies, such as poor adherence to subcutaneous injection and incorrect injection methods (116). In addition, our results indicate that research on PCSK9 inhibitors is limited in developing countries, partly due to the significant economic burden that restricts their clinical use. Therefore, it is crucial to develop other strategies for inhibiting PCSK9 in these situations. Inclisiran, which is still in development and has more persistent effects (54), and even oral peptides (117) may have a chance to solve this problem. Surprisingly, despite the results of three significant Phase III clinical trials of Inclisiran being published in 2020 (54, 118), no Inclisiran-related words were found in our keywords analysis, indicating that research in this field is still in its infancy. The results of studies with cardiovascular outcomes as the endpoint may be highly anticipated. The role of PCSK9 in extrahepatic tissues is largely unknown. Our keywords analysis shows that “inflammation” (**Figure 6B**) and “drug-target mendelian randomization study” (**Figure 8B**) are currently the hotspots and frontiers in this field. This suggests that clinicians and researchers should pay attention to the role of PCSK9 inhibitors in non-cardiovascular fields, such as inflammation, tumors, and the nervous system. In fact, recent Mendelian randomization studies have focused on the associations between PCSK9 inhibition and cognitive function (119), COVID-19 (120), and cancer (121). In addition, the latest research suggests a U-shaped correlation between LDLc levels and the risk of all-cause mortality mediated by infection after acute ischemic stroke (122). This study suggests that even though PCSK9 inhibitors have significant cardiovascular system benefits, further study on cerebrovascular accidents is still required. The role of PCSK9 inhibitors in non-cardiovascular fields is anticipated to be the focus of future research.

### 4.3 Strengths and limitations

The study employs a rigorous approach to comprehend and analyze a substantial amount of data and presents the accumulated scientific findings and subtle evolutionary trends in the area of PCSK9 inhibitors in a visually appealing manner. To our knowledge, this is the first study to analyze the trends in PCSK9 inhibitor research over the past 15 years using the bibliometric tool CiteSpace. Our research aims to lay a solid foundation for future development in this field by assisting clinicians and researchers in quickly obtaining an overview of knowledge in this field and identifying knowledge gaps; identifying potential future research directions and initiating new research ideas; and finally, positioning their expected contributions to the field (123).

However, the present study also has some limitations. First, we only conducted a literature search in the WoSCC database, and relevant studies from other databases may have been missed. Second, only English-language publications were included in our study, so articles published in other languages were missed. These two limitations may lead to biased results.

## 5 Conclusion

This study presents an analysis of research in the field of PCSK9 inhibitors by CiteSpace. Specifically, we performed a visual analysis of items from different years, countries, institutions, authors, keywords, literature, and so on. In conclusion, the number of publications on PCSK9 inhibitors has risen year by year over the past 15 years. The countries, authors, and institutions with the most published articles in the field of PCSK9 inhibitors are from the United States. Compared with developed countries, developing countries such as China need increased international exchange and cooperation. Studies of PCSK9 inhibitors have focused on the mechanisms of LDLc lowering by PCSK9 inhibitors, PCSK9 mAbs and related clinical trials. Coronary artery and inflammation are currently at the forefront of research in the field and are in an explosion period.

## Data availability statement

The original contributions presented in the study are included in the article/supplementary material. Further inquiries can be directed to the corresponding author.

## Author contributions

FL conceived the idea. QL wrote the manuscript. QL and PW collected and read the literature. ZT and ZC conducted the data collection and analysis. ZT, FL and ZF read through and corrected the manuscript. All authors contributed to the article and approved the submitted version.
